# Incidence and Risk Factors for Ipsilateral Shoulder Pain Following Thoracic Surgery

**DOI:** 10.7759/cureus.105238

**Published:** 2026-03-14

**Authors:** Abe Tomoharu, Yuta Mitobe

**Affiliations:** 1 Nursing, Toho University Omori Medical Center, Tokyo, JPN; 2 Perianesthesia Nursing, International University of Health and Welfare, Tokyo, JPN

**Keywords:** ipsilateral shoulder pain, lateral decubitus position, shoulder pain, thoracotomy, video-assisted thoracoscopic surgery

## Abstract

Background

Ipsilateral shoulder pain (ISP) after thoracic surgery is often more difficult to control than incisional pain and may adversely affect postoperative quality of life (QOL).

Objective

This study aimed to determine the incidence of ISP after thoracic surgery and to identify factors associated with its occurrence.

Methods

We conducted a single-center, retrospective observational study including patients aged ≥20 years who underwent thoracic surgery in the lateral decubitus position between April 1, 2022, and March 31, 2024. Patients were classified into an ISP group and a non-ISP group, and perioperative variables were compared between groups.

Results

Of the 328 patients included in the analysis, 116 patients (35.4%) developed ipsilateral shoulder pain (ISP). Logistic regression analysis was performed for variables that showed statistically significant differences. In the univariate analysis, significant differences were observed in 10 variables: height, body mass index (BMI), disease, anesthesia time, operative time, time in the lateral decubitus position, surgical approach, type of surgery, use of epidural anesthesia, and use of postoperative analgesics. These 10 variables were included in the multivariate analysis, which demonstrated that the type of surgery (lobectomy and partial lung resection) and prolonged time in the lateral decubitus position (≥210 minutes) were independently associated with the occurrence of ISP.

Conclusions

The incidence of ISP after thoracic surgery was 35.4%. Prolonged lateral positioning and surgical type may increase the risk of ISP.

## Introduction

Background

Ipsilateral shoulder pain (ISP) in patients undergoing thoracic surgery is often more difficult to manage than incisional pain and can negatively affect postoperative quality of life (QOL). The reported incidence of ISP ranges from 31% to 97%, and ISP may impair postoperative function and rehabilitation [1-3]. ISP is limited to the operated side after thoracotomy, and the severity is reported to be moderate to severe in most patients [1]. ISP typically occurs in the posterior shoulder region and may be felt in the deltoid area, the superoposterior shoulder, or around the lateral third of the clavicle [1].

Reported treatment approaches for ISP include nonsteroidal anti-inflammatory drugs (NSAIDs), acetaminophen, and pregabalin, as well as phrenic nerve block, local infiltration, stellate ganglion block, interscalene brachial plexus block, suprascapular nerve block, and epidural analgesia with catheter placement above the fifth thoracic vertebral level (T5) [1-9].

Multiple unproven or partially proven hypotheses have been proposed regarding the etiology of ISP, including transection of major bronchi, stretching or tension of ligaments due to surgical traction, shoulder joint strain related to intraoperative positioning, pleural irritation from chest drains, and pain due to irritation of the pericardium, mediastinum, or diaphragmatic surface [1]. These mechanisms are broadly categorized into musculoskeletal causes and referred pain mediated by the phrenic nerve [3]. Previous studies have suggested that irritation of the diaphragm and/or structures near the pericardium and mediastinum along the course of the phrenic nerve may contribute to ISP [1,4]; however, no studies have addressed the amount of normal saline used intraoperatively. In thoracic surgery, a large volume of normal saline is often used during leak testing after lung resection and for intrathoracic irrigation. Despite the possibility of direct diaphragmatic stimulation by saline entering the thoracic cavity, this factor has not been investigated.

Objective

This study aimed to identify factors associated with the incidence of ISP in patients undergoing thoracic surgery.

Significance

Although various factors related to ISP have been analyzed in previous studies, no reports have examined the amount of normal saline used intraoperatively. In this study, we added intraoperative normal saline volume and postoperative chest drain output to conventional perioperative variables. Research on ISP remains limited both in Japan and internationally. Accumulating data on factors associated with ISP, as in this study, may contribute to clarifying its mechanisms and establishing effective treatments in the future.

## Materials and methods

Study design

This was a single-center, retrospective observational study.

Participants

**Figure 1:**
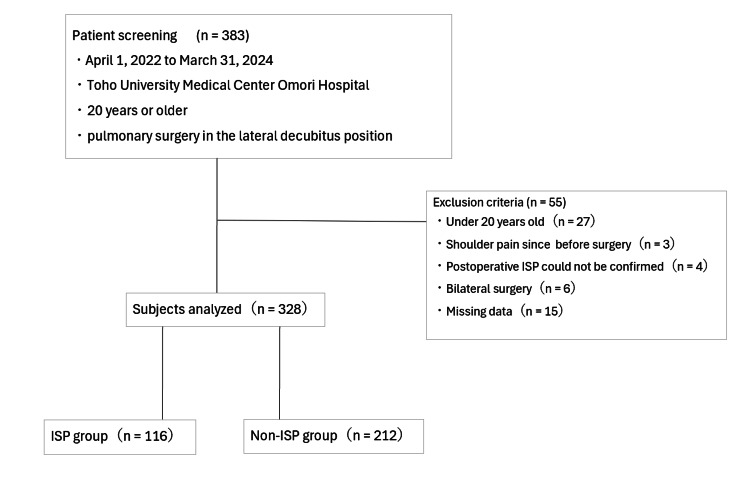
Flowchart of the analyzed study population ISP: ipsilateral shoulder pain

We included patients who underwent thoracic surgery in the lateral decubitus position at Toho University Medical Center Omori Hospital between April 1, 2022, and March 31, 2024. Exclusion criteria were: age <20 years; preexisting shoulder pain; cognitive impairment; inability to confirm the presence or absence of ISP postoperatively; and simultaneous bilateral surgery. The patient selection process is detailed in Figure 1.

Variables and outcome measures

Data were collected from electronic medical records. ISP was defined as postoperative pain from the shoulder to the back that was distinct from incisional pain.

Preoperative Variables

Age, sex, American Society of Anesthesiologists (ASA) Physical Status Classification System [10], height, weight, body mass index (BMI), diagnosis, activities of daily living (ADL), percent predicted vital capacity (%VC), and forced expiratory volume in 1 second (FEV1) were collected.

Intraoperative Variables

Anesthetic technique (inhalational anesthesia or total intravenous anesthesia), operative time, anesthesia time, time in the lateral decubitus position, use of epidural anesthesia, epidural catheter level, surgical approach (thoracotomy, hybrid video-assisted thoracoscopic surgery (hybrid VATS), or complete VATS), type of surgery (partial lung resection, lobectomy (upper/middle/lower lobes; left/right), wedge resection, or others), intraoperative normal saline volume, and intraoperative analgesic use (non-opioid analgesics such as acetaminophen). Opioids were used as part of routine general anesthesia, but were not included as study variables because their use was not standardized.

Postoperative Variables

Presence or absence of ISP, chest drain output, use of analgesics and type of analgesics (NSAIDs and/or acetaminophen), postoperative complications, and postoperative length of hospital stay were collected.

Statistical analysis

Comparisons between the ISP and non-ISP groups were performed using the Mann-Whitney U test and Fisher’s exact test. For continuous variables showing significant between-group differences, receiver operating characteristic (ROC) curve analysis was performed to calculate the area under the curve (AUC) and determine cut-off values; variables were then dichotomized based on these cutoffs. To identify factors associated with ISP, univariate and multivariate logistic regression analyses were conducted with ISP as the dependent variable and variables showing significant intergroup differences as independent variables. All analyses were performed using Easy R (EZR) software, version 1.68 (Department of Hematology, Saitama Medical Center, Jichi Medical University, Shimotsuke, Japan). Statistical significance was set at p < 0.05.

Ethical considerations

This study was approved by the Ethics Committee of Toho University Omori Hospital (approval no. M24073) and the International University of Health and Welfare Group Ethics Review Committee (approval no. 24-Ac-3). An information disclosure document describing the study and allowing participants to opt out at any time was posted on the website of Toho University Medical Center Omori Hospital.

## Results

A total of 383 patients were assessed during the study period, of whom 55 were excluded according to the exclusion criteria. The final analysis included 328 patients, and 116 (35.4%) were classified into the ISP group, as shown in Figure 1.

Preoperative patient characteristics are summarized in Table 1.

**Table 1:** Comparison of ISP group and non-ISP group (preoperative patient background factors) *: Median (minimum,maximum); **: People (%); ADL: activities of daily living; ASA-PS: American Society of Anesthesiology Physical Status; BMI: body mass index; FEV1: forced expiratory volume in 1 second; ISP: ipsilateral shoulder pain; %VC: percent predicted vital capacity

Variables		ISP group (n=116)	Non-ISP group (n=212)	Statistical Analysis	P-Value
Age*	NA	68.50 (21.00, 88.00)	69.00 (20.00, 87.00)	Mann-Whitney U test	0.962
Gender (%)**	Male	66 (56.9)	144 (67.9)	Fisher's exact test	0.054
	Female	50 (43.1)	68 (32.1)	Fisher's exact test	
Height(cm)*	NA	161.10 (138.50, 183.20)	164.20 (138.30, 184.00)	Mann-Whitney U test	0.025
Weight(kg)*	NA	58.95 (33.50, 99.80)	57.70 (36.50, 107.30)	Mann-Whitney U test	0.582
BMI*	NA	23.20 (15.40, 37.00)	22.00 (13.20, 35.20)	Mann-Whitney U test	0.025
ADL (%)**	Independence	116 (100.0)	212 (100.0)	Fisher's exact test	NA
%VC*	NA	103.20 (51.00, 135.00)	100.40 (11.70, 139.60)	Mann-Whitney U test	0.386
FEV1*	NA	76.36 (46.02, 94.40)	75.51 (48.49, 114.20)	Mann-Whitney U test	0.946
ASA-PS (%)**	1	7 (6.0)	28 (13.3)	Fisher's exact test	0.131
	2	84 (72.4)	142 (67.3)	Fisher's exact test	
	3	25 (21.6)	41 (19.4)	Fisher's exact test	

Height and BMI were significantly lower in the ISP group compared with the non-ISP group (Table 1). No significant differences were observed in the other preoperative variables.

Intraoperative factors are summarized in Table 2.

**Table 2:** Comparison of the ISP group and the non-ISP group (intraoperative related factors) *: Median (minimum, maximum); **: People (%); ISP: ipsilateral shoulder pain; T: thoracic; VATS: video-assisted thoracoscopic surgery

Variables		ISP group(n=116)	Non-ISP group(n=212)	Statistical Analysis	P-Value
Disease (%)**	Lung cancer	90 (77.6)	118 (55.7)	Fisher's exact test	<0.001
	Pneumothorax	7 (6.0)	57 (26.9)	Fisher's exact test	
	Others	19 (16.4)	37 (17.5)	Fisher's exact test	
Type of surgery	Partial lung resection	46 (39.7)	52 (24.5)	Fisher's exact test	<0.001
	Lobectomy	49 (42.2)	69 (32.5)	Fisher's exact test	
	Wedge resection	8 (6.9)	59 (27.8)	Fisher's exact test	
	Others	13 (11.2)	32 (15.1)	Fisher's exact test	
Surgical approach (%)**	Thoracotomy	4 (3.4)	5 (2.4)	Fisher's exact test	0.016
	Hybrid VATS	101 (87.1)	162 (76.4)	Fisher's exact test	
	Complete VATS	11 (9.5)	45 (21.2)	Fisher's exact test	
Lateral position orientation**	Right	49 (42.2)	86 (40.6)	Fisher's exact test	0.815
	Left	67 (57.8)	126 (59.4)	Fisher's exact test	
Anesthesia method**	Inhalation anesthesia	73 (62.9)	133 (62.7)	Fisher's exact test	1
	Intravenous anesthesia	43 (37.1)	79 (37.3)	Fisher's exact test	
use of epidural anesthesia (%)**	Yes	104 (89.7)	154 (72.6)	Fisher's exact test	<0.001
	No	12 (10.3)	58 (27.4)	Fisher's exact test	
Epidural level(%)**	T3/4	1 (0.9)	0 (0.0)	Fisher's exact test	NA
	T4/5	3 (2.6)	4 (1.9)	Fisher's exact test	
	T5/6	11 (9.5)	20 (9.4)	Fisher's exact test	
	T6/7	43 (37.1)	72 (34.0)	Fisher's exact test	
	T7/8	34 (29.3)	46 (21.7)	Fisher's exact test	
	T8/9	10 (8.6)	13 (6.1)	Fisher's exact test	
	T9/10	1 (0.9)	2 (0.9)	Fisher's exact test	
	None	13 (11.2)	55 (25.9)	Fisher's exact test	
Operative time(min)*	NA	196.50 (65.00, 657.00)	157.00 (57.00, 457.00)	Mann-Whitney U test	0.042
Time in lateral decubitus position(min)*	NA	224.50 (90.00, 697.00)	184.00 (75.00, 489.00)	Mann-Whitney U test	0.037
Anesthesia time(min)*	NA	272.50 (119.00, 746.00)	233.50 (119.00, 576.00)	Mann-Whitney U test	0.04
Use of intraoperative non-opioid analgesics (acetaminophen)**	Yes	82 (70.7)	156 (73.6)	Fisher's exact test	0.606
	No	34 (29.3)	56 (26.4)	Fisher's exact test	
Amount of saline used during surgery(ml)*	NA	4000.00 (1000.00, 21000.00)	5000.00 (1000.00, 13000.00)	Mann-Whitney U test	0.642

Significant differences were observed between the two groups in diagnosis, type of surgery, surgical approach, and use of epidural anesthesia (Table 2), with higher frequencies in the ISP group. Operative time, anesthesia time, and time in the lateral decubitus position were significantly longer in the ISP group (Table 2). No other intraoperative variables differed significantly.

Postoperative factors are summarized in Table 3.

**Table 3:** Comparison of the ISP group and the non-ISP group (postoperative related factors) *: Median (minimum, maximum); **: People (%); ISP: ipsilateral shoulder pain; NSAIDs: non-steroidal anti-inflammatory drugs

Variables		ISP group (n=116)	Non-ISP group(n=212)	Statistical Analysis	P-Value
Use of postoperative pain medication**	Yes	81 (69.8)	120 (56.6)	Fisher's exact test	0.024
	No	35 (30.2)	92 (43.4)	Fisher's exact test	
Types of postoperative painkillers**	Acetaminophen	68 (58.6)	99 (46.7)	Fisher's exact test	0.057
	NSAIDs	13 (11.2)	21 (9.9)	Fisher's exact test	
	None	35 (30.2)	92 (43.4)	Fisher's exact test	
Postoperative drainage volume(ml)*	NA	180.00 (0.00, 1776.00)	170.00 (0.00, 2150.00)	Mann-Whitney U test	0.373
Postoperative complications**	Yes	7 (6.0)	10 (4.8)	Fisher's exact test	0.612
	No	109 (94.0)	200 (95.2)	Fisher's exact test	
Postoperative hospital stay*	NA	6.00 (3.00, 83.00)	6.00 (2.00, 152.00)	Mann-Whitney U test	0.487

The use of postoperative analgesics was significantly higher in the ISP group compared with the non-ISP group (Table 3). No other postoperative variables showed significant differences.

ROC analysis of preoperative and intraoperative variables

ROC analysis was performed for height and BMI, which showed significant between-group differences. The cut-off value for height was 165 cm (AUC 0.575, 95% CI 0.51-0.64), and that for BMI was 20.5 kg/m² (AUC 0.575, 95% CI 0.51-0.639).

**Figure 2:**
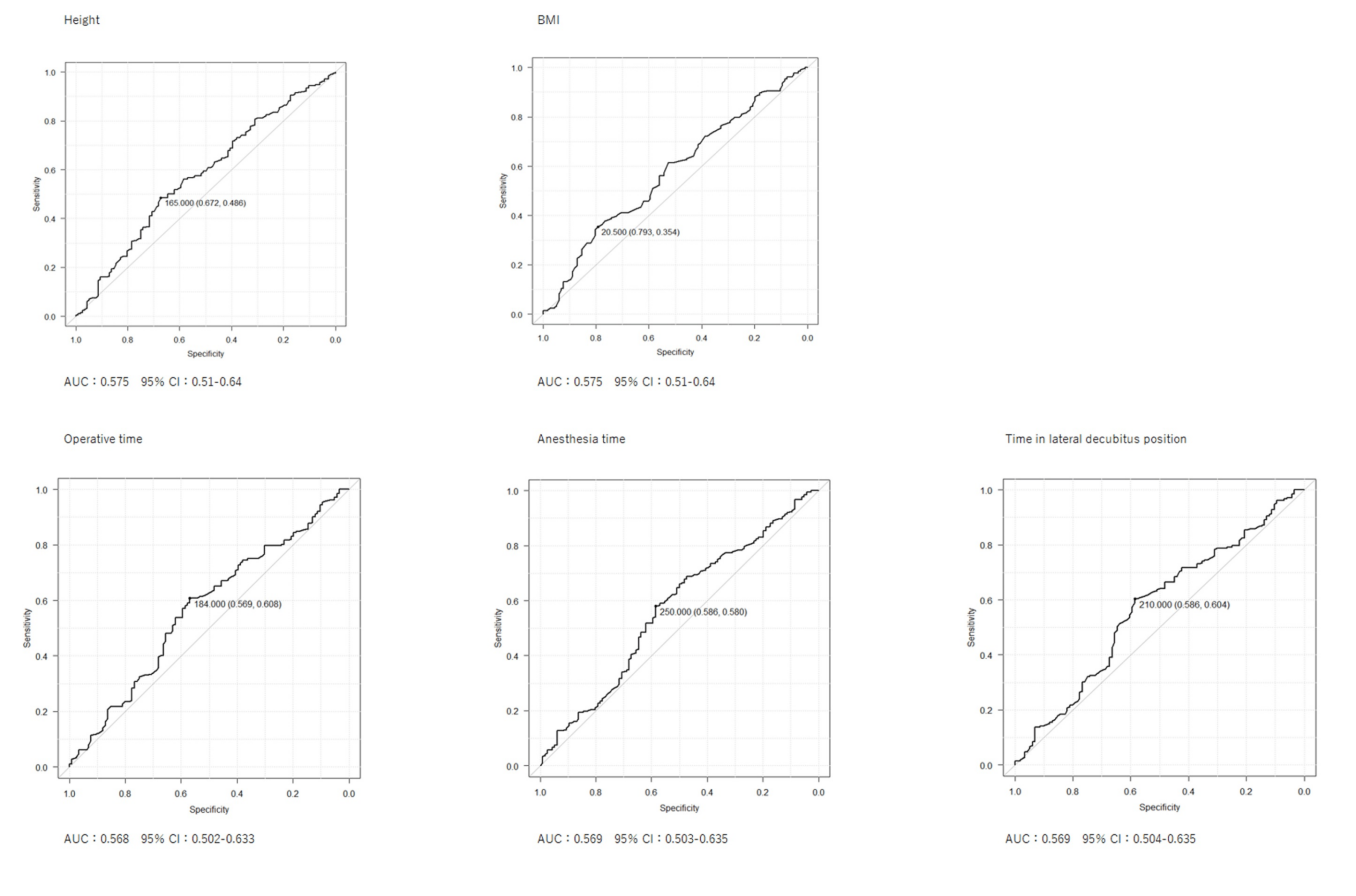
Receiver operating characteristic curves for height, BMI, operative time, anesthesia time, and time in the lateral decubitus position The cut-off value was determined by the maximum degree of the Youden index. AUC: area under the curve; BMI: body mass index

ROC analyses were also performed for operative time, anesthesia time, and time in the lateral decubitus position. The respective cut-off values were 184 min, 250 min, and 210 min. The corresponding ROC curves are shown in Figure 2.

Logistic regression analysis

Variables that showed significant differences in the univariate analysis were entered into logistic regression models. The results of univariate and multivariate logistic regression analyses are presented in Table 4.

**Table 4:** Results of logistic regression analysis for factors associated with the ISP BMI: body mass index; ISP: ipsilateral shoulder pain

Variables	Univariate Analysis			Multivariate Analysis		
	Odds ratio	95%CI	P-Value	Odds ratio	95%CI	P-Value
Height≤165cm	1.87	1.160, 3.010	0.0097	1.62	0.9620, 2.730	0.0697
BMI≤20.5	2.1	1.230, 3.570	0.00614	1.56	0.8740, 2.780	0.133
Disease	0.659	0.479, 0.907	0.0104	1.38	0.8530, 2.240	0.189
Surgical approach	0.459	0.254, 0.829	0.00979	1.16	0.5110, 2.630	0.726
Type of surgery	0.632	0.496, 0.806	0.000223	0.609	0.4230, 0.877	0.00772
Operative time ≥184 (min)	1.97	1.250, 3.120	0.00371	0.333	0.0444, 2.490	0.284
Anesthesia time ≥250 (min)	1.92	1.210, 3.040	0.00533	0.765	0.2120, 2.770	0.683
Time in lateral decubitus position ≥210 (min)	2.12	1.340, 3.350	0.00141	7.52	1.0100, 56.100	0.0493
Use of epidural anesthesia	0.306	0.157, 0.598	0.000534	0.649	0.2710, 1.560	0.333
Use of postoperative analgesics	0.564	0.349, 0.911	0.0194	0.628	0.3750, 1.050	0.0779

In the multivariate analysis, type of surgery (OR 0.609, 95% CI 0.423-0.877; p = 0.00772) and time in the lateral decubitus position (OR 7.52, 95% CI 1.01-56.1; p = 0.0493) remained significantly associated with ISP (Table 4).

Comparisons by diagnosis, type of surgery, and surgical approach

Additional Fisher’s exact tests indicated that operative time ≥184 min, anesthesia time ≥250 min, and time in the lateral decubitus position ≥210 min were associated with a higher risk of ISP. Similarly, height ≤165 cm and BMI ≤20.5 kg/m² were associated with a higher risk of ISP.

**Figure 3:**
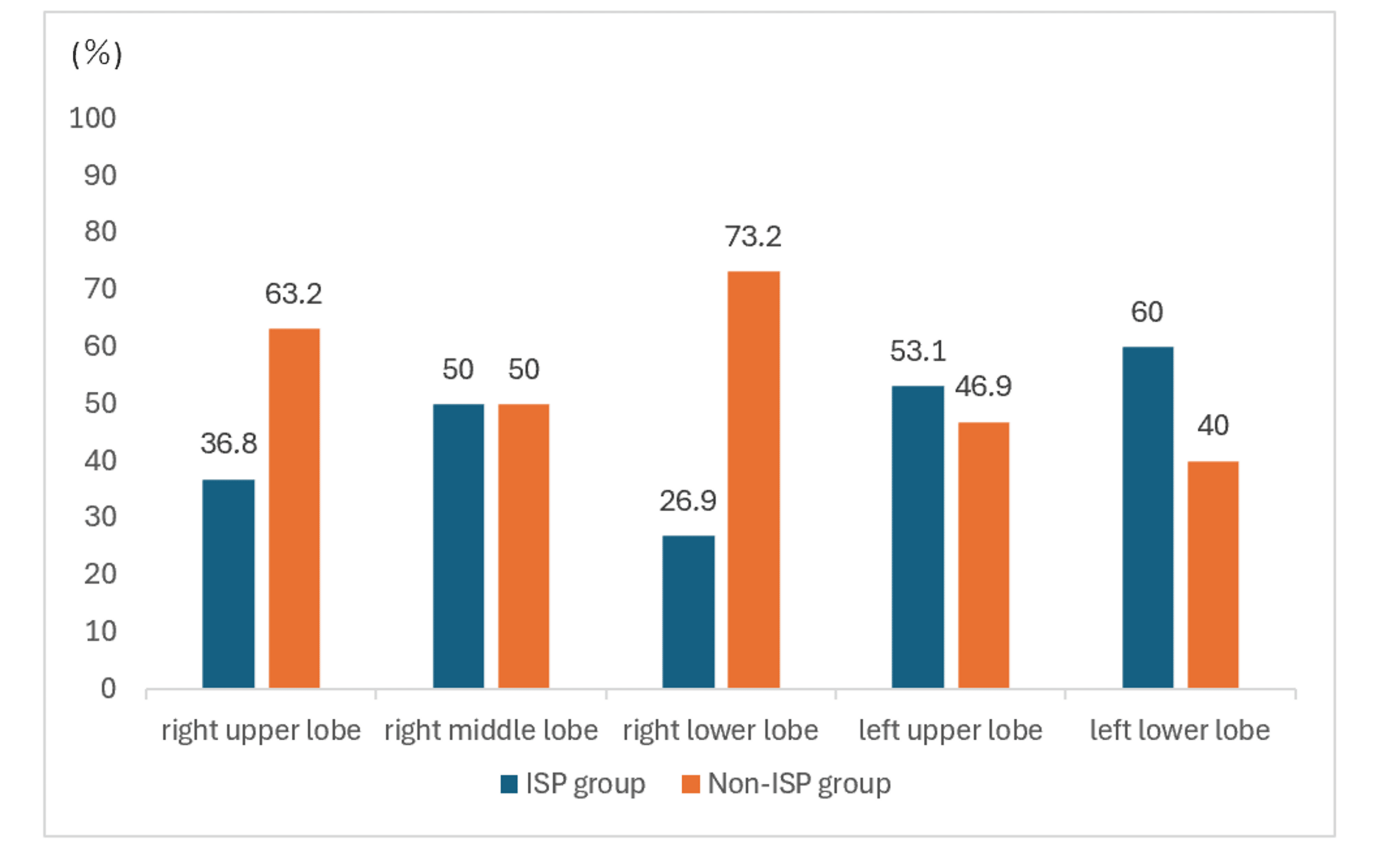
Incidence of ISP by lung lobe ISP: ipsilateral shoulder pain

The incidence of ISP was highest among patients with lung cancer (90 (43.3%)). By type of surgery, partial lung resection showed the highest incidence (46 (46.9%)), followed by lobectomy (49 (40.8%)). Among lobectomy cases, the incidence was higher on the left side. The incidence of ISP according to the lung lobe is shown in Figure 3. Thoracotomy had the highest incidence of ISP (9 (44.4%)), followed by hybrid VATS (263 (38.4%)).

## Discussion

This study investigated the incidence and associated factors of ISP after thoracic surgery. The incidence of ISP was 35.4%. Previous studies have reported ISP incidence rates of 31%-97%, and our findings fall within this range [1,2]. The wide variation across studies may reflect differences in surgical and anesthetic techniques and variations in study populations among institutions.

In this study, height, BMI, diagnosis, surgical approach, type of surgery, operative time, anesthesia time, time in the lateral decubitus position, use of epidural anesthesia, and use of postoperative analgesics were associated with ISP in univariate analyses. In contrast, intraoperative normal saline volume and postoperative chest drain output were not associated with ISP. Previously reported factors associated with ISP include age, operative time, BMI, thoracotomy, epidural catheter level, and upper extremity angle [1,7,11-17].

ISP and intraoperative normal saline volume

Intraoperative normal saline volume (ISP group: 4000 mL vs non-ISP group: 5000 mL; p = 0.642) and postoperative chest drain output (ISP group: 180 mL vs non-ISP group: 170 mL; p = 0.373) were not associated with ISP. One proposed mechanism of ISP is referred pain due to irritation of the pericardium, mediastinum, or diaphragmatic pleural surface mediated by the phrenic nerve. This hypothesis is strongly supported by multiple studies demonstrating the effectiveness of phrenic nerve block (PNB), and by studies in which ISP was induced by phrenic nerve stimulation devices [18]. We hypothesized that physical stimulation of the diaphragm by intraoperative saline could contribute to ISP. However, our results suggest that such stimulation is not associated with ISP, or that the stimulus may be insufficient to trigger ISP. We also assessed the residual intrathoracic saline volume indirectly by postoperative drain output; however, in the supine position, intrathoracic fluid tends to accumulate anatomically toward the lung apex, making diaphragmatic irritation less likely. These findings may explain the lack of association between saline volume and ISP.

ISP and patient factors (body habitus)

We observed that shorter height (≤165 cm) and lower BMI (≤20.5 kg/m²) were associated with ISP. Some previous studies have suggested BMI as a factor related to ISP, reporting higher ISP incidence in patients with higher BMI [7,14]. This has been attributed to tension of the ipsilateral shoulder capsule in the lateral decubitus position, stretching of ligaments, and elongation of periscapular muscles [7,14]. However, our findings indicated a higher incidence of ISP in patients with lower BMI, which contradicts these reports. Another study reported no association between BMI and ISP [19], suggesting that further investigation is needed.

Previous studies have also addressed height. Ren et al. suggested that taller stature is a protective factor against ISP [14]. They proposed that in shorter and obese patients, surgeons may need to increase the degree of arm flexion to secure surgical space, which may increase the risk of ISP. Although their study did not find a significant difference, the high-risk group tended to have shorter height (median 163 cm), consistent with our findings. Although we did not measure shoulder joint angles, shorter height (≤165 cm) may increase the range of motion required of the ipsilateral upper extremity, potentially contributing to somatic pain leading to ISP.

ISP and surgical factors

Prolonged Surgery and Prolonged Positioning

Operative time, anesthesia time, and time in the lateral decubitus position were associated with ISP. Mark et al. suggested that prolonged surgery increases posterior traction on ligaments by the rib retractor, contributing to ISP [10]. Nutchanart et al. identified operative duration >120 min as a factor associated with ISP [12], consistent with our finding of operative time ≥184 min. The cutoffs for anesthesia time ≥250 min and time in the lateral decubitus position ≥210 min may reflect the operative duration; however, time in the lateral decubitus position ≥210 min remained an independent factor in multivariate analysis. Similarly, Sayed et al. suggested that operative time >207.5 min and time in the lateral decubitus position >185 min were associated with higher ISP incidence [13]. Nutchanart et al. noted that prolonged lateral decubitus positioning can impose excessive stress on the shoulder capsule and ligaments, contributing to pain [12]. Longer operations may increase neural stimulation from surgical manipulation and the impact of retractors, while prolonged lateral positioning may increase tension in periscapular muscles and ligaments, potentially promoting ISP.

Surgical Approach and Type of Surgery

Surgical approach and type of surgery were associated with ISP, and type of surgery remained significant in multivariate analysis. The incidence of ISP by approach was 44.4% for thoracotomy (n = 9; ISP 4, non-ISP 5), 38.4% for hybrid VATS (n = 263; ISP 101, non-ISP 162), and 19.6% for complete VATS (n = 56; ISP 11, non-ISP 45). Nutchanart et al. reported thoracotomy as a factor associated with ISP [11]. Sayed et al. also reported a higher incidence of ISP among patients undergoing thoracotomy (45.3%), which is broadly consistent with our findings [13].

In our cohort, hybrid VATS was included as a distinct category. Hybrid VATS involves the use of a rib retractor and is often performed for major lung cancer procedures such as partial lung resection and lobectomy. Anthony et al. reported that even with phrenic nerve infiltration, ISP incidence after hybrid VATS was 60% [19]. In our study, the incidence after hybrid VATS was 38.4%, which is not negligible. While previous studies have largely focused on thoracotomy versus VATS, further research is warranted to clarify the incidence and characteristics of ISP after hybrid VATS.

The incidence of ISP was the highest among patients with lung cancer (43.3%, n = 90), consistent with Sayed et al. [13]. However, in our cohort, lung cancer accounted for 208 of 328 patients (63.4%), which may have influenced this finding. By procedure, partial lung resection showed the highest incidence (47.9%), followed by lobectomy (40.8%), consistent with Burgess and Nutchanart et al. [12,20]. Because time in the lateral decubitus position did not differ substantially among procedures, the higher incidence in partial lung resection and lobectomy may be more closely related to referred pain mediated by the phrenic nerve than to somatic pain from prolonged positioning.

ISP and analgesic strategies

In our study, 69.8% of patients who received epidural anesthesia developed ISP. This supports previous findings suggesting that epidural analgesia may not be effective for preventing ISP [1]. Prior studies have suggested that epidural catheter placement above T5 may be effective [7]; however, no patients in our cohort had catheter placement above T5, and we could not evaluate this association. Scawn et al. reported that 85% of patients receiving epidural analgesia developed ISP, which decreased to 33% with local anesthetic infiltration around the phrenic nerve [4]. Compared with epidural analgesia, stellate ganglion block, interscalene brachial plexus block, phrenic nerve block, and suprascapular nerve block have been reported to be effective for ISP [1,8].

NSAIDs, acetaminophen, and pregabalin have also been reported to be effective for ISP [1]. When ISP occurs, clinicians should assess the severity and characteristics of pain and consider appropriate pharmacologic therapy and/or nerve blocks.

Limitations

This study has several limitations. First, as a single-center retrospective study, the results may be influenced by unmeasured confounding factors. Because the sample was limited to Japanese patients, differences in surgical techniques and body habitus may limit generalizability to other populations. Second, shoulder pain was not assessed using a validated scale, and we did not characterize pain quality or precisely localize pain sites. Although postoperative analgesics were used, we did not assess their effectiveness specifically for ISP. ISP status was determined from chart documentation based on third-party observations, and the exact time of onset of ISP was not consistently recorded. Third, we did not evaluate the position or location of the chest tube, which may influence diaphragmatic or phrenic nerve irritation and could be associated with the development of　ISP. Finally, because this was a retrospective chart review, perioperative management, including positioning details and analgesic protocols, was not completely standardized. Prospective studies with standardized assessments are needed to obtain more accurate data.

## Conclusions

This study aimed to identify factors associated with ISP in patients undergoing thoracic surgery. The incidence of ISP was 35.4%. Type of surgery (lobectomy and partial lung resection) and prolonged time in the lateral decubitus position (≥210 min) were suggested as factors associated with ISP. ISP is a complex, multifactorial phenomenon observed after thoracic surgery. When ISP occurs postoperatively, early therapeutic intervention is necessary to improve postoperative quality of life.
